# *Ampelopsis brevipedunculata* Extract Prevents Bone Loss by Inhibiting Osteoclastogenesis *in Vitro* and *in Vivo*

**DOI:** 10.3390/molecules191118465

**Published:** 2014-11-12

**Authors:** Ju-Young Kim, Sun-Hyang Park, Hyun Mee Oh, Sung Chul Kwak, Jong Min Baek, Myeung Su Lee, Mun Chual Rho, Jaemin Oh

**Affiliations:** 1 Imaging Science-based Lung and Bone Diseases Research Center, Wonkwang University, Iksan, Jeonbuk 570-749, Korea; E-Mails: kimjy1014@gmail.com (J.-Y.K.); ckhlms@wku.ac.kr (M.S.L.); 2 Department of Anatomy, School of Medicine, Wonkwang University, Iksan, Jeonbuk 570-749, Korea; E-Mails: beryls@wku.ac.kr (S.-H.P.); phone8418@hanmail.net (J.M.B.); 3 BK21plus Program & Department of Smart Life-Care Convergence, Graduate School, Wonkwang University, Iksan, Jeonbuk 570-749, Korea; 4 Bioindustrial Process Research Center, Bio-Materials Research Institute, Korea Research Institute of Bioscience and Biotechnology, Jeongeup, Jeonbuk 580-185, Korea; E-Mail:ohhm@kribb.re.kr; 5 Korea Institute of Science and Technology for Eastern Medicine (KISTEM), NeuMed Inc., Seoul 130-831, Korea; E-Mail: Ksc960@naver.com; 6 Division of Rheumatology, Department of Internal Medicine, Wonkwang University, Iksan, Jeonbuk 570-749, Korea; 7 Institute for Skeletal Disease, Wonkwang University, Iksan, Jeonbuk 570-749, Korea

**Keywords:** *Ampelopsis brevipedunculata* extract (ABE), bone loss, bone resorption, osteoclast differentiation

## Abstract

Osteoclasts play a critical role in bone resorbing disorders such as osteoporosis, periodontitis, and rheumatoid arthritis. Therefore, discovery of agents capable of suppressing osteoclast differentiation may aid the development of a therapeutic access for the treatment of pathological bone loss. *Ampelopsis brevipedunculata* has been used as herbal folk medicine to treat liver diseases and inflammation in Asia. However, its effects on osteoclast differentiation are unknown. We were aimed to investigate the anti-osteoclastogenic activity *in vitro* and *in vivo* and to elucidate the underlying mechanism of *Ampelopsis brevipedunculata* extract (ABE). In this study, ABE inhibited receptor activator of NF-κB ligand (RANKL)-induced osteoclast differentiation, the formation of filamentous actin rings and the bone resorbing activity of mature osteoclasts. ABE inhibited RANKL-induced p38 and IκB phosphorylation and IκB degradation. Also, ABE suppressed the mRNA and protein expression of nuclear factor of activated T cells c1 (NFATc1) and c-Fos, and the mRNA expression of genes required for cell fusion and bone resorption, such as *osteoclast-associated* *receptor (OSCAR)*, *tartrate resistant acid phosphatase (TRAP)*, *cathepsin* *K*, *dendritic cell-specific transmembrane protein* *(DC-STAMP)*, *β3-integrin* and *osteoclast stimulatory transmembrane protein* *(OC-STAMP)*. Furthermore, results of micro-CT and histologic analysis indicated that ABE remarkably prevented lipopolysaccharide (LPS)-induced bone erosion. These results demonstrate that ABE prevents LPS-induced bone erosion through inhibition of osteoclast differentiation and function, suggesting the promise of ABE as a potential cure for various osteoclast-associated bone diseases.

## 1. Introduction

Bone is a very dynamic organ that is maintained by a delicate balance between osteoclast-mediated bone destruction and osteoblast-mediated bone formation. This balance can be disturbed by over-activation of osteoclasts, resulting in bone destruction and fragile bones. Osteoporosis is a critical problem characterized by bone loss and impaired bone quality that can lead to an increased risk of fracture. Bone fracture results in increased mortality in elderly people [1,2]. Therefore, control of bone loss is a key way for preserving the quality of life of elderly patients suffering from disorders related to excessive bone resorption. 

Osteoclasts are bone-resorbing multinucleated giant cells derived from hematopoietic stem cells. The receptor activator of NF-kappa B ligand (RANKL), a member of the tumor necrosis factor (TNF) family, promotes formation of osteoclasts from osteoclast precursors in the presence of macrophage-colony stimulating factor (M-CSF) [3,4]. Binding of RANKL to its receptor RANK activates multiple downstream signaling pathways, including NF-κB and mitogen-activated protein (MAP) kinases, and activates major transcription factor nuclear factor of activated T cells c1 (NFATc1) [5]. NFATc1 induces the expression of key osteoclastogenesis-related molecules that are required for successful osteoclast differentiation, including *osteoclast-associated receptor (OSCAR)*, *tartrate resistant acid phosphatase (TRAP)*, *cathepsin K*, *dendritic cell-specific transmembrane protein* *(DC-STAMP)*, *β3-integrin* and *osteoclast stimulatory transmembrane protein* *(OC-STAMP)* [6,7,8,9]. 

Several natural products exert inhibitory effects on osteoclast differentiation and function, leading to suppressed bone loss *in vivo*. Examples from available literature include grape-seed proanthocyanidin extract, *Bacillus***-**fermented antler extract, and *Aconitum pseudo-laeve var. erectum* extract [10,11,12]. To discover new compounds that can act as anti-resorption agents, we screened natural products that regulate osteoclast differentiation and identified the extract of *Ampelopsis brevipedunculata* (ABE) as one such agent. ABE has anti-inflammatory and anti-hepatotoxic activities [13,14,15]. However, the effects of ABE on RANKL-induced osteoclast differentiation have not been studied. 

In the present study, we investigated the effects of ABE on the signaling pathways involved in osteoclast differentiation and activation, as well as the *in vivo* effect of ABE in a lipopolysaccharide (LPS)-induced bone erosion mice model. 

## 2. Results and Discussion

### 2.1. ABE Suppresses RANKL-Mediated Osteoclast Differentiation in Bone Marrow Derived Macrophages (BMMs)

**Figure 1:**
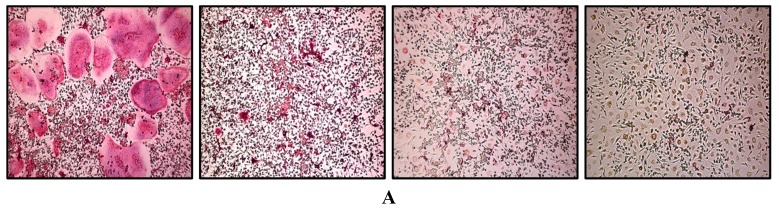

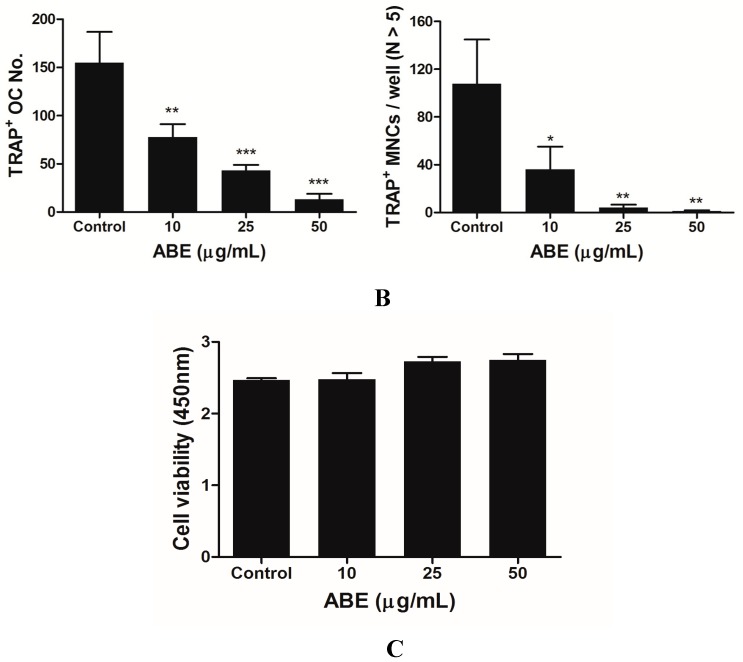
Effect on osteoclast differentiation by ABE. (**A**) BMMs were cultured for 4 days with M-CSF (30 ng/mL) and RANKL (100 ng/mL) in the presence or absence of ABE. Cells were fixed in 3.7% formalin, permeabilized with 0.1% Triton X-100, and stained with TRAP solution. TRAP-positive cells were photographed under a light microscope. (**B**) TRAP-positive cells were counted as osteoclasts (left). TRAP-positive multinucleated osteoclasts (TRAP^+^ MNCs) with more than five nuclei were counted. * *P* < 0.05, ** *P* < 0.01, *** *P* < 0.001 *vs.* control. (**C**) BMMs were seeded into a 96-well plate and cultured for 3 days in the presence of M-CSF (30 ng/mL) and with the indicated concentrations of ABE. After 3 days, the absorbance was measured at 450 nm using an ELISA reader.

To investigate the effects of ABE on RANKL-mediated osteoclastogenesis, we treated primary BMMs in the presence of RANKL and M-CSF with or without various concentrations of ABE. While RANKL differentiated the BMMs of the control group into TRAP-positive mature multinucleated osteoclasts, ABE decreased the formation of TRAP-positive multinucleated cells in a dose-dependent manner (Figure 1A,B). Next, to investigate whether suppressed osteoclastogenesis by ABE was due to the potential toxicity of this product, we also examined the cytotoxicity of ABE by XTT. ABE had no cytotoxic effects at doses that effectively inhibited osteoclast differentiation (Figure 1C).

### 2.2. ABE Inhibits RANKL-Mediated Phosphorylation of p38 and IκB in BMMs 

**Figure 2:**
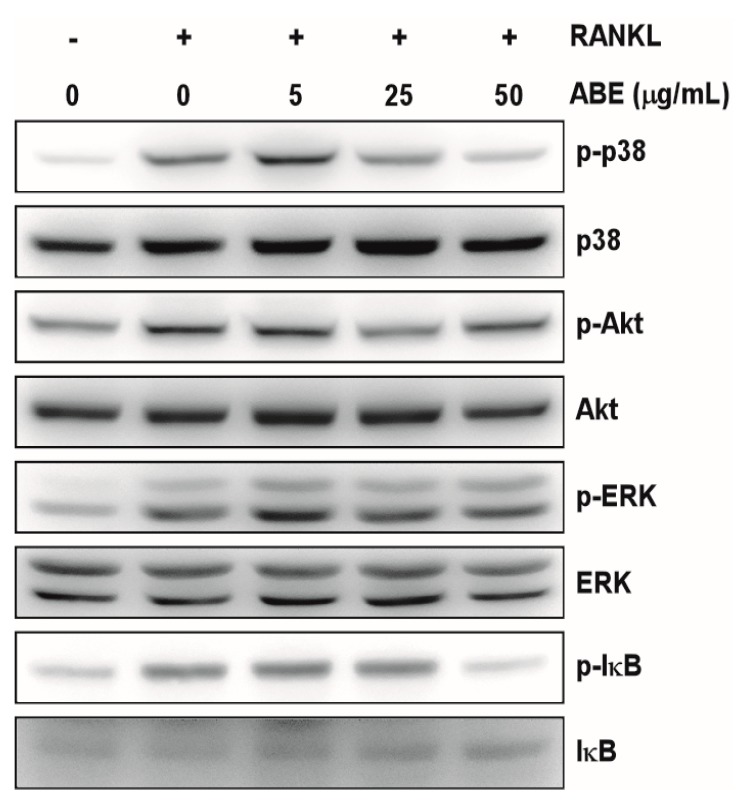
Effect of ABE on RANKL-induced early signaling pathway. BMMs were cultured for 1 day in the presence of M-CSF (10 ng/mL). After, BMMs were starved for 3 h, pretreated with ABE for 1 h in the indicated concentrations and then stimulated with RANKL (100 ng/mL) for 7 min. Cell lysates were analyzed by western blotting with antibody against p-p38, p38, p-Akt, Akt, p-ERK, ERK, p-IκB, and IκB.

Binding of RANKL to its receptor RANK activates a chain of major intracellular signaling, including MAP kinases, NF-κB, and Akt. Several studies have reported that MAP kinases, composed of ERK and p38, are involved in RANKL-induced osteoclast differentiation [16,17]. Therefore, we investigated the effect of ABE on RANKL-induced signaling pathways to understand the mechanism underlying the ABE-mediated inhibition of RANKL-induced osteoclast differentiation. After the osteoclast precursors were pretreated with various concentrations of ABE for 1 h and stimulated with RANKL for 7 min, we observed several signaling pathways and found that phosphorylation of p38 and IκB by RANKL were significantly down-regulated by ABE (Figure 2). The p38 signaling pathway plays a key role in bone destruction, and therefore, is considered a potential therapeutic target for bone-destructive diseases [18,19]. NF-κB is an important transcription factor for RANKL-activated osteoclastogenesis [20]. NF-κB moves into the nucleus after the phosphorylation of IκB by IκB kinase. In this process, the interaction between IκB and NF-κB is achieved through blocking the function of the nuclear localization sequence (NLS) [21]. Severe osteopetrosis and defects in osteoclast differentiation are observed in NF-κB-knockout mice, indicating that NF-κB is an important factor in osteoclastogenesis. Our data suggest that ABE suppressed RANKL-mediated osteoclastogenesis through inhibition of p38 and NF-κB. Based on the previous studies, we suggest possible approaches that ABE induces NF-κB inactivation *via* malfunctioning NLS promoter region.

### 2.3. ABE Inhibits the RANKL-Induced Expression of NFATc1 in BMMs 

**Figure 3:**
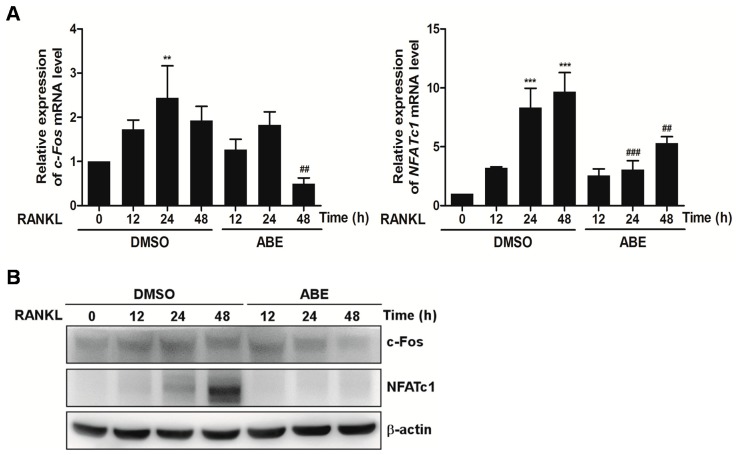
Effect of ABE on RANKL-induced c-Fos and NFATc1 expression. (**A**) BMMs were pretreated with ABE (50 μg/mL) for 1 h and then stimulated with M-CSF (30 ng/mL) and RANKL (100 ng/mL) for the indicated times. Total RNA of c-Fos and NFATc1 were obtained at the indicated time points. The mRNA expression levels of the indicated genes were analyzed by quantitative real-time RT-PCR. (**B**) BMMs were pretreated with or without ABE (50 μg/mL) for 1 h and then stimulated with M-CSF (30 ng/mL) and RANKL (100 ng/mL) for the indicated times. The cell lysates were analyzed by western blotting with c-Fos, NFATc1, and β-actin antibodies. ** *P* < 0.01, *** *P* < 0.001 *vs.* control; ^##^ *P* < 0.01, ^###^ *P* < 0.001 *vs.* DMSO treated group in the indicated time, respectively.

Since NFATc1 plays essential roles in osteoclast differentiation, we investigated the effects of ABE on the expression of NFATc1. Osteoclast precursors were treated with ABE and further stimulated with RANKL at indicated time points. We found that the mRNA and protein levels of c-Fos and NFATc1 increased in response to RANKL, and that the increased c-Fos and NFATc1 expression was significantly inhibited by ABE (Figure 3A,B). Ectopic expression of NFATc1 transforms the precursors of osteoclasts to multinucleated mature osteoclasts even in the absence of RANKL. Furthermore, embryonic stem cells with NFATc1 deficit do not successfully differentiate into osteoclasts in response to RANKL, suggesting an essential role of NFATc1 in osteoclastogenesis [22]. During RANKL-induced osteoclast differentiation, the expression of NFATc1 is controlled by two promoters, P1 and P2. P1 that represents a DNase I hypersensitive chromatin site and contains several sites for binding of transcription factors is known to induce expression of isoform A of NFATc1. Another promoter, P2 is also known to derive expressions of isoform B and C of NFATc1 [23,24]. NFATc1 is also induced by the p38 signaling pathway. Therefore, the suppressive effect of ABE on osteoclastogenesis could result from its potential ability to induce the inactivation of NFATc1 through targeting P1 and P2 promoters and inhibit the p38-NFATc1 signaling axis.

### 2.4. ABE Inhibits mRNA Expression of OSCAR, TRAP, Cathepsin K, DC-STAMP, β3-Integrin and OC-STAMP by RANKL 

**Figure 4:**
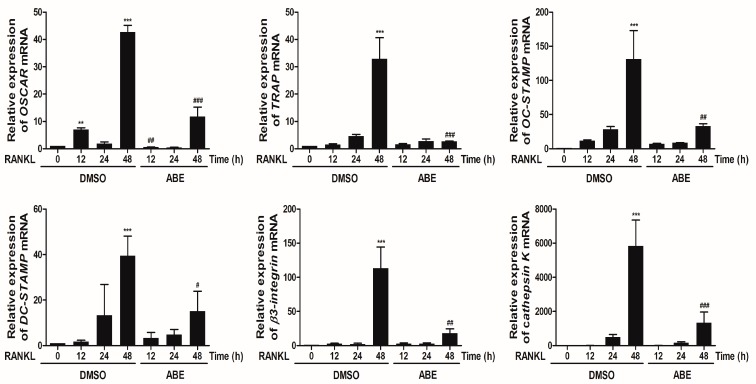
Effect of ABE on RANKL-mediated expression of osteoclast-related genes. BMMs were pretreated with or without of ABE (50 μg/mL) for 1 h and then treated with M-CSF (30 ng/mL) and RANKL (100 ng/mL) for the indicated time points. Total RNA from the cells was obtained and the expression of *OSCAR, TRAP, cathepsin K*, *DC-STAMP*, *β3-integrin* and *OC-STAMP* mRNA was analyzed by quantitative real-time RT-PCR. ** *P* < 0.01, *** *P* < 0.001 *vs.* control; ^#^ *P* < 0.05, ^##^ *P* < 0.01, ^###^ *P* < 0.001 *vs.* DMSO treated group in the indicated time, respectively.

NFATc1 plays an essential role in the regulation of genes involved in osteoclast differentiation. NFATc1 is expressed in the middle or late stages of osteoclast differentiation mediated by RANKL, and it subsequently induces the expression of osteoclast-specific genes, including *OSCAR*, *TRAP*, *cathepsin K*, *DC-STAMP*, *β3-integrin* and *OC-STAMP* [6,7,8,9]. Excessive bone resorption is a key event that leads to pathological bone destruction and fragility. Bone resorption is performed by osteoclasts, which are specialized cells capable of solubilizing collagen, a component of bone matrix. Cathepsin K, a cysteine proteinase, has been identified as the major proteinase responsible for the degradation of collagen by osteoclasts and induced by NFATc1 [25,26]. Also, *ανβ3-integrin* is known to play a role in the regulation of cell migration and the maintenance of the sealing zone required for effective osteoclastic bone resorption [27]. We examined whether ABE can regulate the expression of *OSCAR*, *TRAP*, *cathepsin K*, *DC-STAMP*, *β3-integrin* and *OC-STAMP* that play essential roles during RANKL-mediated osteoclastogenesis. ABE inhibited the mRNA expression of *OSCAR*, *TRAP*, *cathepsin K*, *DC-STAMP*, *β3-integrin* and *OC-STAMP* by RANKL (Figure 4). These data indicate that ABE may inhibit RANKL-induced mRNA expression of *OSCAR*, *TRAP*, *cathepsin K*, *DC-STAMP*, *β3-integrin* and *OC-STAMP* through suppression of NFATc1.

### 2.5. ABE Inhibited the Formation of F-Actin Rings and Bone Resorption Activity of Mature Osteoclasts in Vitro

**Figure 5:**
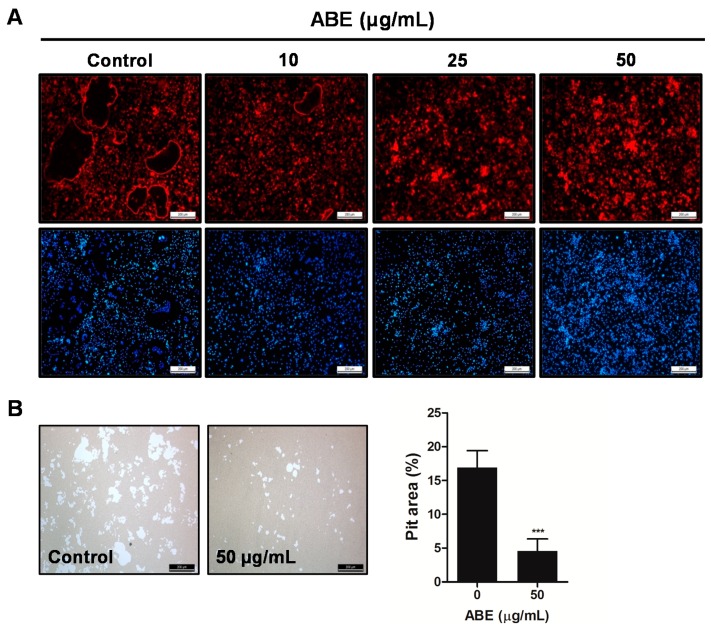
Effect of ABE on bone resorbing activity of mature osteoclasts. (**A**) Phalloidin and DAPI staining of osteoclasts treated with or without ABE. (**B**) Mature osteoclasts were seeded on hydroxyapatite-coated plates and treated for 24 h with the indicated concentrations of ABE. Attached cells on the plates were removed and photographed under a light microscope. Pit areas on hydroxyapatite plate were quantified using the Image Pro-PLUS (Ver. 4.5) software. *** *P* < 0.001 versus the control.

We next examined whether ABE has the potential to inhibit the formation of F-actin rings and bone resorption activity of mature osteoclasts. The formation of F-actin rings was distinctly apparent in BMMs treated with DMSO (as a control). However, the differentiation of BMMs treated with ABE into mature osteoclasts with F-actin structure was limited in a dose-dependent manner (Figure 5A). Also, after mature osteoclasts were cultured on hydroxyapatite-coated plates in the presence or absence of ABE for 24 h, we observed the resorbed area under a microscope. ABE decreased the area of the resorption pits created by mature osteoclasts, while the control could not suppress the increase in the number of resorption pits being generated, suggesting that ABE inhibits the bone resorption activity of mature osteoclasts (Figure 5B). These results suggest that ABE suppresses the formation of F-actin ring structures and the bone resorption activity of mature osteoclasts.

### 2.6. ABE Prevented LPS-Mediated Bone Destruction in Vivo 

**Figure 6:**
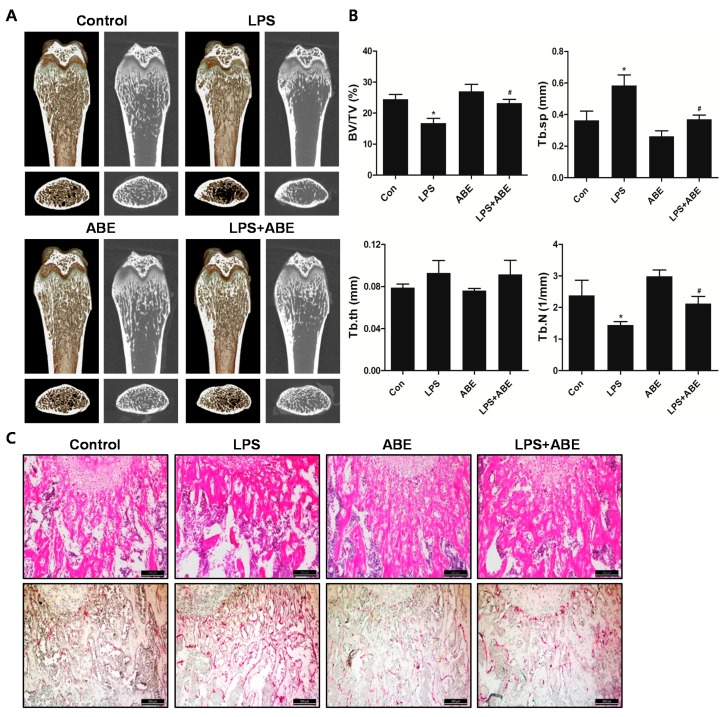
Effect of ABE on LPS-mediated bone erosion. (**A**) Mice were sacrificed on day 10, and radiographs of the longitudinal and transverse section of a 2- or 3-dimensional visualization of the proximal femurs were obtained with a micro-CT apparatus. (**B**) The trabecular bone volume/total volume (BV/TV), trabecular separation (Tb.Sp), trabecular thickness (Tb.Th), and trabecular number (Tb.N) of the femurs were determined using the micro-CT data and INFINITT-Xelis software. * *P* < 0.05 the control group, ^#^ *P* < 0.05 the LPS group. (**C**) Dissected femora were fixed, decalcified, embedded, and sectioned. Sections were stained with H&E (upper) and were stained with TRAP (lower).

To examine the effect of ABE on *in vivo* bone loss, LPS-induced bone erosion mouse model was chosen as previously described by our group. Mice were intraperitoneally injected with LPS with or without ABE. The mice were sacrificed on day 10 and the left femurs underwent micro-computed tomography (micro-CT) analyses. A visualization of the femoral area revealed the massive loss of trabecular bone following LPS treatment. On the other hand, LPS-induced bone loss was clearly reduced in the femurs of ABE-treated and LPS-injected (Figure 6A). Morphometric analyses of the femurs from LPS-treated mice revealed pronounced reductions in bone volume per tissue volume (BV/TV) and Trabecular number (Tb.N) along with an increase in trabecular separation (Tb.Sp), we observed that the reduction of BV/TV and Tb.N along with the increase of Tb.Sp following the LPS injection was recovered in the ABE-treated, LPS-induced mice (Figure 6B). Histological analysis showed that the LPS-induced osteoclast formation and bone loss were greatly inhibited in the femurs of ABE-treated mice (Figure 6C). The results suggest that ABE suppresses bone loss via pre-osteoclast fusion and osteoclast activity, while LPS induces the production of inflammatory factors, supporting the survival of mature osteoclasts and stimulating osteoclastic bone resorption.

## 3. Experimental Section 

### 3.1. Ethanol Extract of ABE 

The dried stems and roots of *Ampelopsis brevipedunculata* were purchased from an herbal store in seoul, Korea. The plant materials (9 kg) were extracted with EtOH for 7 days at room temperature. The EtOH extract was passed through a 0.45 µm filter and vaporized in a rotary evaporator, yielding 250 g of residue. The dried EtOH extract (250 g) was suspended in distilled water and then diluted with culture medium to the desired working concentration. 

### 3.2. Mice and Reagents 

Male, 5-week-old ICR mice were purchased from Damul Science (Daejeon, Korea) and housed in controlled temperature (22–24 °C) and humidity (55%–60%) with 12 h light/dark cycles. All experiments in this study were performed in accordance with the animal experiment guidelines of the Institute Committee of Wonkwang University (Iksan, Korea). Recombinant soluble human M-CSF and human RANKL were obtained from PeproTech EC Ltd. (London, UK). Anti-ERK 1/2, anti-phospho-ERK 1/2, anti-p38, and anti-phospho-p38, anti-Akt, and anti-phospho-Akt, anti-IκB, and anti-phospho-IκB antibodies were purchased from Cell Signaling Technology Inc. (Beverly, MA, USA). Anti-c-Fos and anti-NFATc1 antibodies were purchased from Santa Cruz Biotechnology (Santa Cruz, CA, USA). Monoclonal β-actin antibody was obtained from Sigma (St. Louis, MO, USA). Fetal bovine serum (FBS), α-minimum essential medium (α-MEM), and penicillin/streptomycin were purchased from Gibco BRL (Grand Island, NY, USA). 

### 3.3. Mouse Bone Marrow Macrophage Preparation and Osteoclast Differentiation 

Bone marrow cells were obtained by flushing the femurs and tibiae of 5-week-old ICR mice with α-MEM and suspended in α-MEM supplemented with 10% FBS. Non-adherent cells were collected and cultured for 3 days in the presence of M-CSF (30 ng/mL). Floating cells were discarded and adherent cells on dish bottoms were classified as BMMs. BMMs were seeded at 3.5 × 10^4^ cells/well in α-MEM/10% FBS and cultured in the presence of M-CSF (30 ng/mL) and RANKL (100 ng/mL) for 4 days in the presence or absence of ABE. Osteoclasts were identified by staining for TRAP activity. 

### 3.4. Cytotoxicity Assay Western blot Analysis, Quantitative Real-time RT-PCR Analysis

XTT assay, western blot analysis, and quantitative real-time RT-PCR were performed as described previously [28]. 

### 3.5. Actin Ring Staining 

BMMs were cultured for 3 days with M-CSF (30 ng/mL) and RANKL (100 ng/mL) in the presence or absence of ABE. Cells were fixed with PBS containing 3.7% formaldehyde and permeabilized with PBS containing 0.1% Triton-X-100. The cells were blocked 2.5% bovine serum albumin (BSA) and incubated with phalloidin (Molecular Probes, Eugene, OR, USA) at room temperature for 30 min, washed and rinsed with PBS before mounting with DAPI (Sigma, Steinheim, Germany). The images were taken using a fluorescence microscope (DMLB, Leica, Wetzlar, Germany). 

### 3.6. Resorption Pit Assay 

Mature osteoclasts were prepared by isolating osteoblasts from the calvariae of newborn mice by serial digestion in collagenase (Wako, Osaka, Japan), as previously described [28]. Bone marrow cells were isolated as described above. Osteoblasts and bone marrow cells were co-cultured on a collagen gel-coated 90-mm dish in the presence of 1α, 25-dihydroxyvitamin D_3_ (VitD_3_) and prostaglandin E_2_ (PGE_2_) for 6 days. Co-cultured cells were detached by treatment with 0.2% collagenase at 37 °C for 10 min, were re-plated on hydroxyapatite-coated plates and incubated with or without ABE for 24 h. After 24 h, the cells were removed, and total resorption pit areas were photographed and analyzed by the Image Pro-Plus program version 4.0 (Media Cybernetics, Rockville, MD, USA).

### 3.7. In Vivo LPS-Induced Bone Loss 

To examine the effect of ABE on *in vivo* bone destruction, ICR mice (5 weeks old) were divided into four groups of 5 mice. Mice were administered orally ABE (150 mg/kg body weight) or PBS as control 1 day before injection of LPS (5 mg/kg body weight). ABE or PBS (control) was administered orally every other day for 9 days. The mice received intraperitoneal injections of LPS or PBS on days 2 and 6. All mice were sacrificed on day 10. The left femurs underwent high-resolution micro-CT analysis. BV/TV, Tb.sp, trabecular thickness (Tb.th) and Tb.N. were applied to perform quantitiative analysis using INFINITT-Xelis software (INFINITT Healthcare, Seoul, Korea). The femurs were fixed in 4% paraformaldehyde (Sigma) for 1 day, decalcified for 3 weeks in 12% ethylenediaminetetraacetic acid (EDTA), and embedded in paraffin. Sections of 5 mm thickness were prepared using a Leica microtome RM2145 (Leica Microsystems, Bannockburn, IL, USA). For histologic examination, sections were stained with hematoxylin and eosin (H&E), and other sections were stained with TRAP to identify osteoclasts on the bone surface.

### 3.8. Statistical Analysis 

Each experiment was performed at least three times and all quantitative data are presented as mean ± standard deviation (SD). All statistical analyses were performed using SPSS (Korean version 14.0, IBM, Armonk, NY, USA). Student’s *t*-test was used to compare the parameters between two groups, while the analysis of variance (ANOVA) test, followed by the Tukey *post-hoc* test was used to compare the parameters among 3 groups. *P* < 0.05 was considered statistically significant.

## 4. Conclusions 

Our findings clearly show that ABE has an antiosteoclastogenic potential by reducing the *in vitro* RANKL-induced p38, NF-κB, c-Fos and NFATc1 in osteoclast. Moreover, ABE also prevented *in vivo* LPS-induced bone destruction. Thus, our findings strongly indicate that ABE deserves new evaluation as a potential treatment option in various bone diseases associated with excessive osteoclast formation and bone destruction.
